# Anti-Biofilm Extracts and Molecules from the Marine Environment

**DOI:** 10.3390/md22070313

**Published:** 2024-07-10

**Authors:** Flore Caudal, Catherine Roullier, Sophie Rodrigues, Alain Dufour, Sébastien Artigaud, Gwenaelle Le Blay, Alexis Bazire, Sylvain Petek

**Affiliations:** 1 Laboratoire de Biotechnologie et Chimie Marines, Université Bretagne Sud, EMR CNRS 6076, IUEM, 56100 Lorient, France; flore.caudal@univ-ubs.fr (F.C.); sophie.rodrigues@univ-ubs.fr (S.R.); alain.dufour@univ-ubs.fr (A.D.); 2 IRD, Univ Brest, CNRS, Ifremer, LEMAR, IUEM, 29280 Plouzane, France; sebastien.artigaud@univ-brest.fr (S.A.); gwenaelle.leblay@univ-brest.fr (G.L.B.); 3 Institut des Substances et Organismes de la Mer, Nantes Université, ISOMER, UR 2160, 40000 Nantes, France; catherine.roullier@univ-nantes.fr

**Keywords:** biofilm, marine natural products, bacteria, anti-biofilm

## Abstract

Pathogenic bacteria and their biofilms are involved in many diseases and represent a major public health problem, including the development of antibiotic resistance. These biofilms are known to cause chronic infections for which conventional antibiotic treatments are often ineffective. The search for new molecules and innovative solutions to combat these pathogens and their biofilms has therefore become an urgent need. The use of molecules with anti-biofilm activity would be a potential solution to these problems. The marine world is rich in micro- and macro-organisms capable of producing secondary metabolites with original skeletons. An interest in the chemical strategies used by some of these organisms to regulate and/or protect themselves against pathogenic bacteria and their biofilms could lead to the development of bioinspired, eco-responsible solutions. Through this original review, we listed and sorted the various molecules and extracts from marine organisms that have been described in the literature as having strictly anti-biofilm activity, without bactericidal activity.

## 1. Introduction

Antimicrobial resistance (AMR) is a global public health problem, limiting therapeutic strategies against bacterial infections. The World Health Organization (WHO) has identified this issue as a priority, as it is estimated that drug-resistant infections contributed to nearly 5 million deaths in 2019 [1]. The Organization for Economic Cooperation and Development (OECD) indicates that resistance to antibiotics of last resort could increase twofold by 2035, highlighting the urgent need for robust antimicrobial stewardship practices and also for increased research of novel compounds.

The situation could be even worse, as most studies of AMR fail to take into account that bacteria often adopt a biofilm lifestyle in their environment and when causing infections. Bacterial biofilms are communities of cells located at interfaces, embedded into a self-produced matrix composed of exopolysaccharides, proteins, lipids, and extracellular DNA. The matrix contributes to protection from the environment by providing a relatively impermeable physical barrier to toxic substances, including antibiotics [2]. The multilayered organization of cells enhances this protection, as only peripheral cells are usually exposed to external agents [3]. Persistent cells found in the deeper layers of the biofilm are less sensitive to antibiotics, due to their mechanism of action which generally targets actively growing bacteria [4].

The overuse of antibiotics leads to an increase in their concentration in the environment of human activities. Their increasing concentration is problematic because they have been shown to induce biofilm formation, leading to an adaptive response of bacteria [5,6,7] and potentially to gene transfer from animal to human pathogens [8].

Cell proximity in biofilm structures allows two types of communication between bacteria: (i) genetic, through horizontal gene transfer favoring the exchange of antibiotic resistance genes [9], and (ii) chemical, through the perception of small molecules, allowing the estimation of population density to perform joint actions, a phenomenon known as quorum sensing (QS) [10]. QS-controlled phenotypes include bacterial virulence and regulation of biofilm formation, and QS is therefore an interesting therapeutic target, as its short-circuiting (quorum quenching) can impair biofilm formation and/or reduce virulence mechanisms [11].

The discovery of new molecules and innovative solutions to prevent biofilm formation or disrupt biofilms of pathogenic bacteria has become critical. The use of molecules with anti-biofilm activity, inhibiting the pathways that regulate virulence and biofilm formation, would be a potential solution to these problems, rather than trying to eradicate bacteria through the use of “traditional” antibiotics.

For many years, research in the pharmaceutical industry focused primarily on natural products from the terrestrial world, which were generally easier to access. However, in the middle of the 20th century, technological and technical advances in diving and remotely operated vehicles such as ROVs (remotely operated underwater vehicles), made it easier to explore the marine world [12], with the promise of new bioactive molecules, as 70% of Earth’s surface is covered by oceans and seas.

The marine world is teeming with micro- and macro-organisms capable of producing secondary metabolites with original skeletons and interesting activities. Numerous research teams are aware of this, and are interested in the chemical strategies used by some of these organisms to regulate and/or protect themselves from pathogenic bacteria and their biofilms, in order to develop bioinspired, eco-responsible solutions. Several strategies are used by organisms to limit pathogenic bacterial biofilms, including inhibiting microbial growth, interfering with bacterial communication, disrupting adhesion processes, or destroying pre-formed biofilms, as in the case of matrix polymer-degrading enzymes.

We focused this review on articles describing molecules or extracts from marine organisms with specific anti-biofilm or anti-QS activities. This approach is quite original compared to other existing reviews, as we avoided all molecules with antibacterial properties that consequently prevent the establishment of biofilm. Using “molecules”, “biofilm”, “antibiofilm”, “anti-biofilm”, and “marine” as keywords for our PubMed^®^ search, we found 71 articles that met this criterion between 2009 and 2023. Many articles were excluded because they also reported anti-biofilm molecules with antibacterial activities. We decided not to include these results because of a potential bias: if a certain proportion of bacteria is killed, then less biofilm is formed, making it difficult to conclude that the molecule is strictly anti-biofilm.

The first section presents the active extracts for which the active molecule was not found or isolated, followed by a presentation of the purified and identified molecules. Finally, the various results reported here are discussed.

## 2. Anti-Biofilm Compounds and Quorum-Sensing Inhibitors

Among the different articles dealing with pure anti-biofilm activities (without antibacterial activity), we found 20 articles presenting the activity of extracts and supernatants, and 51 articles presenting the results of purified molecules from marine organisms. These will be treated separately as they imply different issues and development perspectives.

### 2.1. Extracts and Culture Supernatants

Several publications described extracts or culture supernatants of macro- or micro-organisms with anti-biofilm activities, for which the active molecules have not yet been described. From a review of these articles, the most common models for biofilms were those from *Staphylococcus aureus* and *Pseudomonas aeruginosa*, two pathogens with major public health issues. Indeed, they are involved in numerous multi-resistant chronic infections. In most cases, the nature of the active molecules in these extracts or culture supernatants was not identified. This may be explained by the fact that, in some cases, purification leads to activity loss as several compounds in the mixture might have synergistic effects. Therefore, culture supernatants are directly tested or only after a first round of purification to separate organic and aqueous fractions.

Bakkiyaraj et al. used methanolic extracts from *Streptomyces akiyoshiensis*, an actinomycete associated with the coral *Acropora digitifera*, against various strains of *S. aureus*, including methicillin-resistant strains and or clinical strains. These extracts showed anti-biofilm activity at MBIC = 0.1 mg/mL (MBIC: Minimum Biofilm Inhibiting Concentration) and was able to inhibit intestinal colonization in the nematode *Caenorhabditis elegans* [13].

Leetanasaksakul et al. showed an anti-biofilm activity against *S. aureus* and *Escherichia coli* biofilms from 13 and 10 marine actinomycetes culture supernatants, respectively, out of 101. Interestingly, those that were active on *E. coli* biofilm were not active on *S. aureus* and vice versa. They showed a significant reduction of more than 60% of the biofilm. Analysis of the culture supernatants showed that most actinomycetes secrete non-toxic anti-biofilms metabolites with varying degrees of proteolytic activity. Non-toxicity towards bacteria is an important feature, as it prevents them from developing resistance. Out of the 23 active culture supernatants, only 4 also showed antibacterial activity [14].

The fungus *Blastobotrys parvus* PPR3 isolated from a mangrove wood sample (*Avicennia marina*) also showed promising activity. The crude extract of PPR3 reduced various virulence characteristics of *P. aeruginosa*, in particular pyocyanin, elastase, protease, and chitinase production, as well as motility, biofilm formation, exopolysaccharide, and alginate production. The authors were able to demonstrate an interaction with *P. aeruginosa* LuxR type receptors, suggesting an inhibition of QS [15].

Extracts derived from three algae, *Ulva lactuca*, *Halopteris scoparia* (ex *Stypocaulon scoparium*), and *Pterocladiella capillacea*, were prepared by successive macerations with different solvents (cyclohexane, dichloromethane, ethyl acetate, and methanol). Extracts obtained with cyclohexane and ethyl acetate showed *P. aeruginosa* biofilm inhibitory activity, but with different mechanisms of action [16]. In a second study, the team looked at the effect of the same extracts on *S. aureus.* Here the four extracts showed inhibition in *S. aureus* biofilm formation, with action on adhesion and proliferation stages [17].

From a red seaweed, *Gracilaria changii*, Muthukrishnan et al. showed a strong anti-biofilm and anti-QS activity against *Vibrio campbellii* BB120. The crude methanol extract showed activity at 1 µg/mL, with a decrease in biofilm formation and inhibition of violacein production by *C. violaceum* [18].

Still based on seaweed extracts, this time from three different algae, *Chaetomorpha aerea*, *Agardhiella subulata*, and *Hypnea cornuta*, anti-biofilm activities were searched for against diverse marine pathogens. The tests on different *Vibrio* species and *Listonella anguillarum* showed anti-adhesive properties of the extracts with modification of hydrophobicity levels and cell surface charges. They also demonstrated the lack of toxicity of these extracts on aquaculture [19].

Wang et al. exhibited some interesting anti-biofilm activity from several extracts of coastal mangroves of Mayotte against a clinical strain of *P. aeruginosa*. Three of the twenty-three extracts showed more than 50% inhibition of biofilm formation [20].

A really interesting review of marine algae-derived anti-biofilm compounds by Behzadnia et al. showed that, with these different extracts and the molecule that will be present in the next section, and the one they presented, algae should be a really promising source of anti-biofilm compounds. These compounds could be very useful for human, animal, and environmental health [21].

Using methanolic extracts of different parts (tentacle, disc, and whole body) of Haddon’s sea anemone, *Stichodactyla haddoni*, collected in the Persian Gulf, Hamayeli et al. showed a predominance of aliphatic compounds with anti-biofilm activity against *Bacillus cereus* and *P. aeruginosa* [22].

In 2021, the same team extracted metabolites from two sponges, *Psammocinia* sp. and *Hyattella* sp., using a mix of two organic solvents, and tested their anti-biofilm activity against six bacteria: *P. aeruginosa*, *Acinetobacter baumannii*, *Klebsiella pneumoniae*, *E. coli*, *S. aureus*, and *B. cereus*. Both extracts showed significant effects, probably due to the presence of phenolic compounds, butanedioic acid, propanoic acid, and benzene-acetaldehyde, without however identifying the active molecule(s) [23].

Methanolic extract of the sponge *Agelas dispar* was shown to inhibit biofilm formation and destroy biofilm of *Candida krusei* (ATCC6258), *C. glabrata* (ATCC 2001), and *C. parapsilosis*. It appears that this extract causes changes in the cytoplasmic membrane and/or changes in the cell wall [24].

Various sponge extracts isolated from Wallis were tested for their anti-biofilm activities, particularly against the marine pathogen *Vibrio harveyi* ORM4. Twenty-eight different genera were tested and seven of them showed anti-biofilm activities. Four different extracts from the genus *Hyrtios* were among the most efficient with up to 93.61% inhibition of biofilm formation [25].

Some studies came very close to identifying an active molecule. Balasubramanian et al. (2017) were able to demonstrate the activity of *Streptomyces* sp. SBT343, a sponge-associated actinomycete, on different strains of *Staphylococcus* [26]. In their subsequent study, they succeeded in purifying the SKC3 compound and carried out initial characterization works, but without obtaining the exact structure. At concentrations ranging between 3.95 and 31.25 µg/mL, SKC3 inhibited *S. epidermidis* biofilm formation. Analysis of the transcriptome of treated bacteria revealed a negative effect on central metabolism, notably carbon flux, but also amino acid, lipid, and energy metabolism [27].

Bacteria belonging to the *Pseudoalteromonas* genus are sources of numerous anti-biofilm metabolites, identified or not. The culture supernatant of *Pseudoalteromonas haloplanktis* TAC125, isolated in Antarctica, inhibits the biofilm of *S. epidermidis* [28]. The mode of action has not yet been fully elucidated, but the molecule is suspected to act as an AI-2 agonist or as a ligand targeting the AI-2 receptor, AI-2 being a universal language for interspecies communication. Moreover, the molecule appears to be produced at all stages of bacterial growth and under a wide variety of experimental conditions [29]. The team subsequently succeeded in identifying a pentadecanal, a long-chain fatty aldehyde, which acts on the AI-2 pathway [30], and then tested the activity of derivatives of this pentadecanal and showed an increase in activity with pentadecanoic acid [31].

The same research team, still using Antarctic marine bacteria, showed anti-biofilm activity on ESKAPE bacteria, which are a major health issue. Interestingly, these four culture supernatants did not exhibit any antimicrobial activity but acted on biofilm formation and pre-formed biofilms, mainly of *S. aureus*, *K. pneumoniae*, and *P. aeruginosa* [32].

Among 86 heterotrophic marine bacteria, Doghri et al. identified the *Pseudomonas* sp. IV2006 strain, the culture supernatant of which inhibited the biofilm of another marine bacterium, *Flavobacterium* sp. II2003. The supernatant altered the surface properties of the glass, making it more hydrophilic and alkaline, thus significantly reducing bacterial adhesion. The supernatant was also active against biofilms of human pathogens such as *S. aureus*, *P. aeruginosa*, and *Yersina enterocolitica* [33].

Enzymes are another family of molecules with the potential to perform interesting activities. An interesting activity of a stony coral, *Montipora foliosa*, a supernatant on the pathogen *Stenothrophomonas maltophilia*, was shown in the article by Peters et al. A group of metalloproteases responsible for anti-biofilm activity was identified by proteomic analysis of this active supernatant [34].

This first section on active extracts or culture supernatants highlights the diversity of their origins in the marine world, whether from macro-organisms such as sponges or anemones, or from micro-organisms such as algae, bacteria, or fungi. The study of marine diversity is therefore a promising avenue for research of active natural compounds.

Most of the teams have not yet gone as far as to purify the active molecule, but this section includes very recent papers, published in the last five years, and the rest of the story may not yet have been published or is likely still in progress. There are a number of additional factors that can complicate further studies, such as the limited availability of molecules, particularly those extracted from marine macro-organisms, which may be available in limited quantities, or the non-homogeneous production of metabolites by a micro-organism, depending on the culture conditions.

The use of culture supernatants or extracts saves time in the search for anti-biofilm molecules. In fact, this use can be seen as a screening to see where the anti-biofilm molecules are. Bio-guided purification can then be used to move from the fractions to the active molecule in a more or less timely manner. This saves a lot of time compared to purifying and then testing every single molecule produced by an organism. Extracts from sponges are also readily available and are usually made from freeze-dried material, allowing their chemical diversity to be studied and conserved. For bacteria and algae, culture supernatants are often reproducible and available in larger quantities.

However, the demonstration of activity in an extract or culture supernatant does not necessarily mean that the active molecule will be easy to purify and characterize. In fact, it often turns out that this activity may be due to several molecules in the extract or culture supernatant, or to a synergy of molecules that lose their activity once separated. Extracts from sponges or cnidarians are often available in limited quantities, so characterization of the active molecule(s) may be hampered by the problem of accessing larger quantities.

The second section of this review describes the identified molecules that have been isolated and characterized from marine organisms and that have anti-biofilm and/or anti-QS activities.

### 2.2. Active Compounds

**Figure 1:**
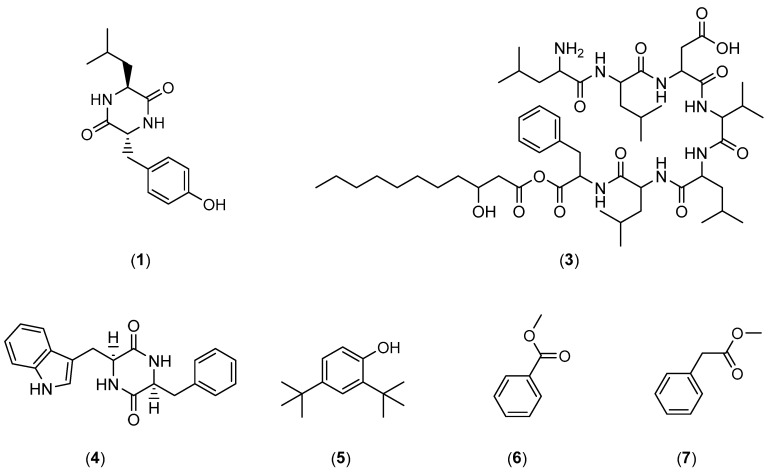
Chemical structures of cis-cyclo(Leucyl-Tyrosyl) (**1**), nesfactin (**3**), cyclo(*L*-Trp-*L*-Ser) (**4**), 2,4-di-tert-butylphenol (**5**), methyl benzoate (**6**), and methyl phenylacetate (**7**).

**Figure 2:**
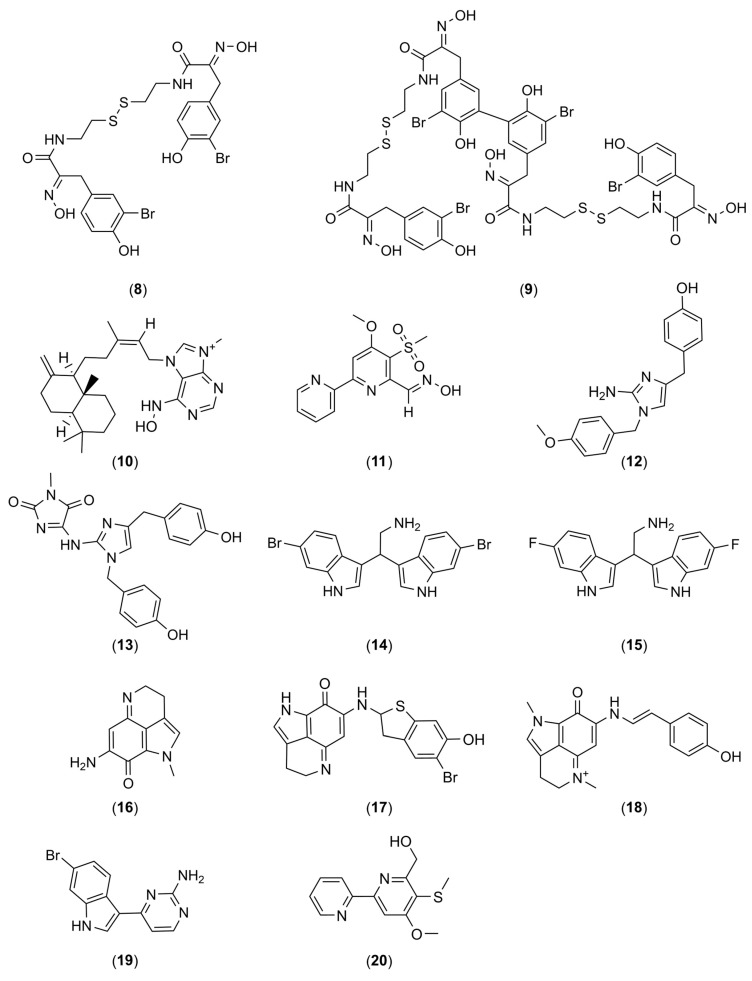
Chemical structures of psammaplin A (**8**), bisaprasin (**9**), ageloxime D (**10**), maipomycin A (**11**), isonaamine D (**12**), isonaamidine A (**13**), 2,2-*bis*(6-bromo-1*H*-indol-3-yl)ethanamine (**14**), 2,2-*bis*(6-fluoro-1*H*-indol-3-yl)ethanamine (**15**), makaluvamine A (**16**), makaluvamine F (**17**), makaluvamine G (**18**), meridianin D (**19**), and collismycin C (**20**).

**Figure 3:**
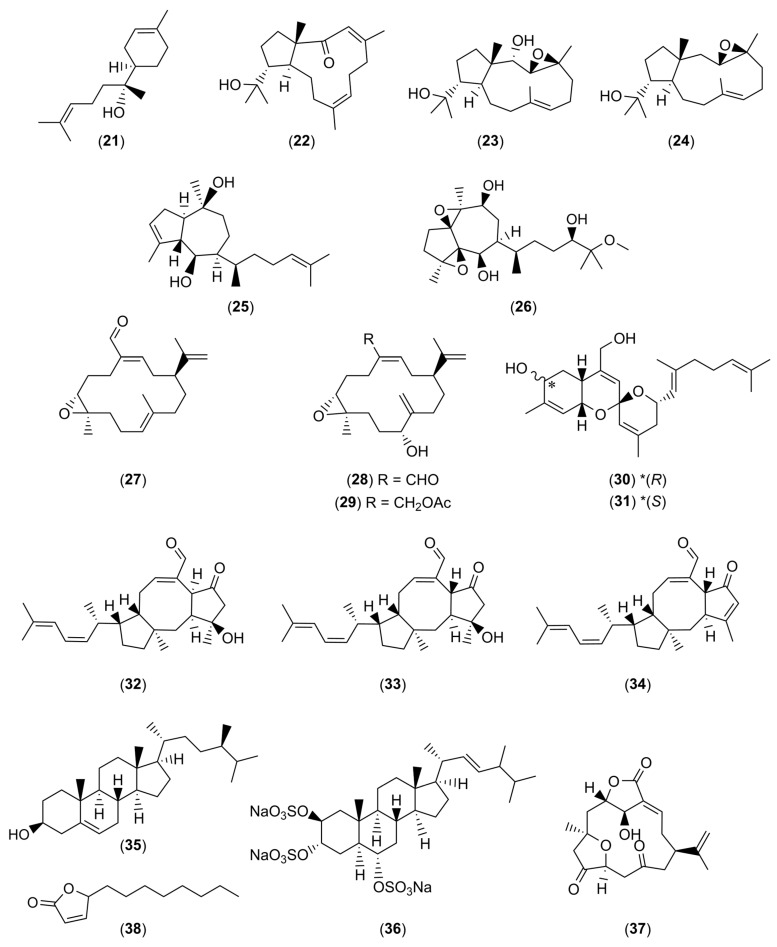
Chemical structures α-bisabolol (**21**), three dolabellanes (**22**–**24**), dictyol C (**25**), dictyol L (**26**), knightal (**27**), 11(R)-hydroxy-12(20)-en-knightal (**28**), 11(R)-hydroxy-12(20)-en-knightol acetate (**29**), phorbaketal B (**30**), phorbaketal C (**31**), ophiobolin K (**32**), 6-epi-ophiobolin K (**33**), 6-epi-ophiobolin G (**34**), siphonocholin (**35**), halistanol sulfate A (**36**), 5-episinuleptolide (**37**), and 5-octylfuran-2(5H)-one (**38**).

**Figure 4:**
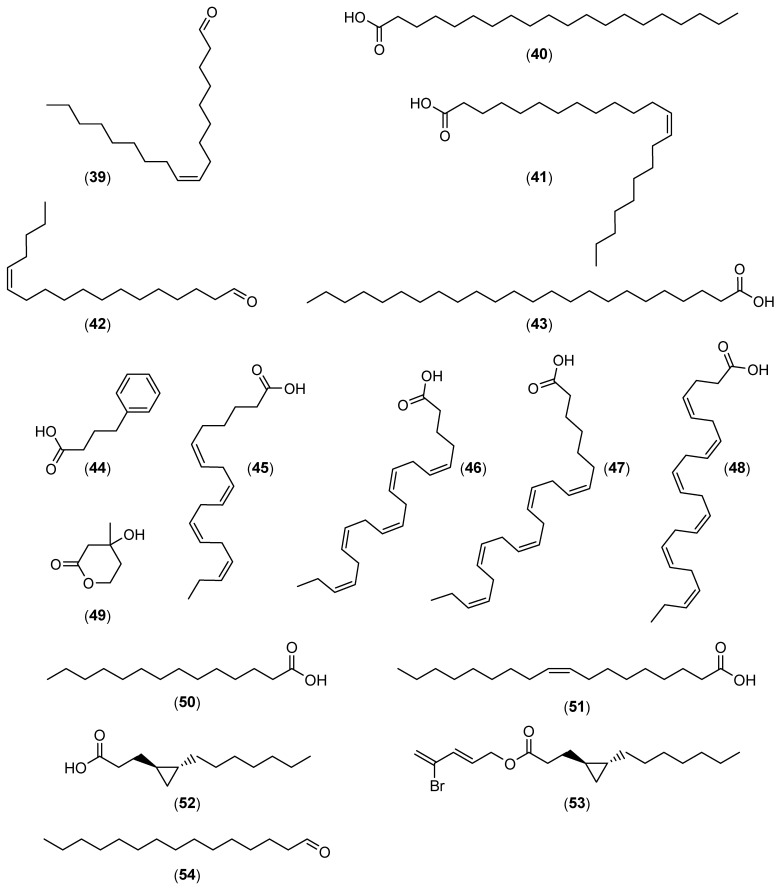
Chemical structures of (9Z)-9-octadecenal (**39**), arachic acid (**40**), erucic acid (**41**), (13Z)-13-octadecenale (**42**), tetracosanoic acid (**43**), 4-Phenylbutanoic acid (**44**), stearidonic acid (18:4 n-3) (**45**), eicosapentaenoic acid (20:5 n-3) (**46**), docosapentaenoic acid (22:5 n-3) (**47**), docosahexaenoic acid (22:6 n-3) (**48**), mevalonolactone (**49**), myristic acid (**50**), oleic acid (**51**), lyngbyoic acid (**52**), benderadienne (**53**), and pentadecanal (**54**).

**Figure 5:**
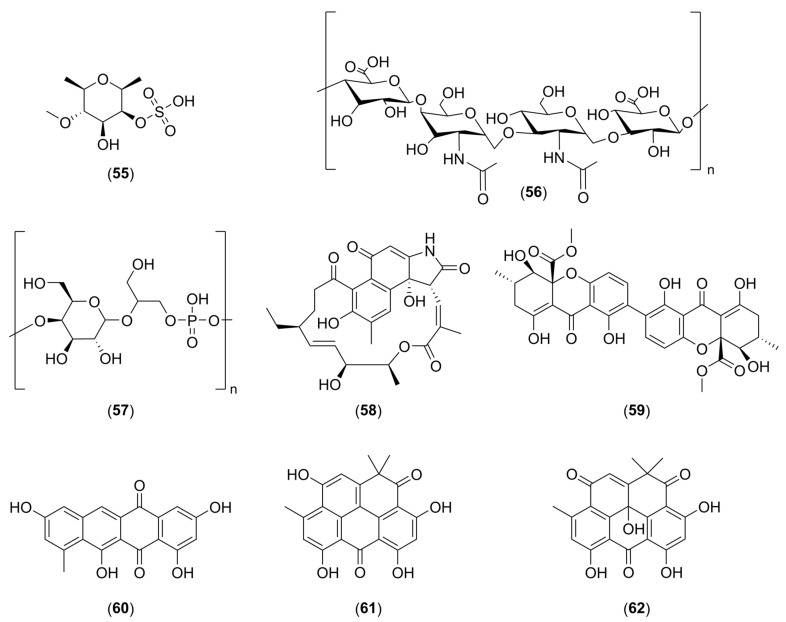
Chemical structures of fucoidan (**55**), MO245 (**56**), α-D-galactopyranosyl-(1→2)-glycerol-phosphate (**57**), hygrocin C (**58**), secalonic acid D (**59**), tetracenomycin D (**60**), resistomycin (**61**), and resistoflavin (**62**).

In addition to the numerous interesting extracts described in the literature, it is possible to find more or less purified molecules whose modes of action have sometimes been demonstrated. Table 1 describes the various non-biocidal molecules found in the literature. The compounds are numbered and their structures are shown in Figure 1, Figure 2, Figure 3, Figure 4 and Figure 5.

As with culture supernatants and extracts, the diversity of producing organisms is notable.

This table includes seven different families of molecules: peptides and proteins, phenolic compounds, alkaloids, terpenoids, fatty acids and derivatives, polysaccharides, and polyketides. The target micro-organisms mainly studied are of the *Pseudomonas* or *Staphylococcus* genus. The mode of action is specified when it is described in the articles, but it is not always known precisely.

## 3. Discussion

Through our review, we showed a great diversity of anti-biofilm molecules produced in the marine world: peptides and proteins, phenolic compounds, alkaloids, terpenoids, fatty acids and derivatives, and polyketides. We also showed that many marine organisms are potential producers of anti-biofilm molecules: bacteria, fungi, algae, and invertebrates (sponges, corals, echinoderm, mollusks, ascidians, etc.). Active supernatants or extracts have also been determined, for which the active molecule(s) have not yet been identified.

Bacteria are the most studied, accounting for nearly 40% of the producers studied, probably due to their abundance in the environment and also because they represent a convenient renewable and sustainable resource to exploit. Access to larger quantities of molecules is facilitated. In terms of ecology, they are in constant competition with each other to occupy environmental niches crucial for their survival, which probably explains their great capacity to produce anti-biofilm molecules. Marine fungi also compete with bacteria and therefore have the ability to inhibit biofilm, accounting for nearly 15% of identified producers.

Filter-feeding organisms such as sponges and mollusks are also widely studied, as they are in constant contact with bacteria. Sponges are really interesting, especially regarding the intriguing chemical skeletons of their metabolites. From the point of view of anti-biofilm research, sponges have the advantage of being sessile organisms, producing numerous metabolites that enable them to control the bacteria that colonize them, probably by repelling some via anti-biofilm metabolites or attracting others for symbiosis [85].

Finally, some organisms, such as algae, have developed strategies to avoid colonization of their thallus, also making them ideal sites to search for anti-biofilm compounds.

For many macro-organisms, however, it is their association with bacteria to form the so-called holobiont that is probably at the origin of the production of active metabolites. Thus, the part played by bacterial metabolites is certainly largely underestimated, and it is often difficult to know whether it is one of the two organisms that produces the molecule, or whether it is the association of the two that makes it possible. Cultivation of bacteria isolated from macro-organisms is therefore no guarantee of success in recovering activity.

Of the sixty molecules tested, just over a third had activity on several pathogens, which is all the more interesting given that biofilms are generally mixed with at least two types of bacteria. The other molecules should not be ruled out, as they may not yet have been tested on other pathogens.

**Figure 6:**
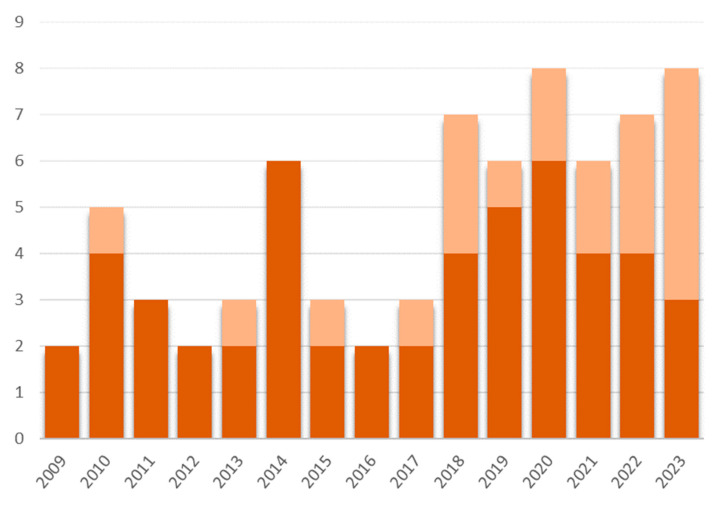
Number of publications reviewed in the present study, by year, presenting extracts (pale orange) or pure molecules (bright orange) with strictly anti-biofilm action.

Figure 6, which groups together the years of publication of the articles reviewed in this study, also shows the recent interest in this field.

Most studies on anti-biofilm activities of marine natural resources have been published in the last 15 years with more than 60% of articles after 2016 (Figure 6). The most recent articles even present extracts or culture supernatants for which the molecules have not yet been described.

Despite the fact that these compounds are of marine origin, the majority of the authors have tested their activities against biofilms of human pathogenic bacteria such as *E. coli*, *Salmonella*, and *Streptococcus mutans*, and in 50% of cases against *P. aeruginosa* and *S. aureus*, which are models for bacterial biofilm studies. The most targeted marine bacterial genus is *Vibrio*, to which belong the main pathogens in marine environments.

*P. aeruginosa* and *Staphylococcus* were chosen because of the wealth of data available on these strains and because they are a major problem in health care. In fact, they are used as models in many areas of research, and the QS in *P. aeruginosa* was one of the first to be described, along with that of *Vibrio*. Moreover, a large amount of molecular data on biofilm formation mechanisms is available, making it easier to understand the mechanisms of action of the identified anti-biofilm molecules.

To take our synthesis work one step further, we decided to compare the molecules using Datawarrior ^®^ software version 5.5.0 [86]. The structural similarities were first studied and the clusters identified logically represented the different classes of molecules described. However, no structural similarities were shown, which could not explain their shared anti-biofilm activities. This can be explained by the diversity of modes of action that anti-biofilm molecules can have. In fact, even though they are grouped under a single name, their actions can be totally different. As mentioned earlier, they can affect bacterial communication, disrupt the adhesion process, or degrade matrix polymers.

Based on their structures, by calculating different parameters of these molecules such as logP or Druglikeness (DL) score, those presenting the best potential for drug development could be highlighted. In fact, logP is a good indicator of bioavailability of molecules in the human body. As an example, for good oral absorption, values of less than 5 are usually preferred. The Druglikeness score is based on the presence of different fragments of the molecule compared to a collection of fragments of commercial drugs and compounds. A positive score indicates that the molecule under investigation contains mostly fragments found in marketed drugs. Out of the 50 molecules described, only 8 have a clogP of less than 5 and a positive Druglikeness score. Toxicity tests are not always performed in studies, but could be used to discriminate between a larger number of candidate drugs. It is important to note that logP is included in the calculation of the Druglikeness score, but sometimes the LD score is reduced by the lipophilicity of certain molecules. However, with current galenic formulation techniques, and depending on the intended application, these molecules should not be excluded (Table 2).

However, these assumptions are theoretical and would require laboratory testing to determine the actual toxicity of the compounds. Indeed, among the eight molecules that could be considered as potential best candidate drugs, there are the makaluvamines, which are nonetheless known for their toxicity [87,88].

**Table 2:** Analysis of the logP values and Druglikeness scores of the molecules using Datawarrior^®^ software version 5.5.0. (In blue: molecules with logP < 5 and Druglikeness score > 0).

Compound Family.	Compound Number	Compound Name	LogP	Druglikeness Score
Peptides and proteins	**1**	*cis*-cyclo(Leucyl-Tyrosyl)	**1.1773**	**4.294**
	**2**	Paracentrin 1	/	/
	**3**	Nesfactin	4.0566	−31.67
	**4**	Cyclo(*L*-Trp-*L*-Ser)	1.8772	4.4232
Phenolic compounds	**5**	2,4-di-*tert*-butylphenol	4.4777	−5.276
	**6**	Methyl benzoate	1.5726	−3.9278
	**7**	Methyl phenylacetate	1.5707	−6.9825
Alkaloids	**8**	Psammaplin A	**4.2446**	**1.5181**
	**9**	Bisaprasin	8.4888	1.5181
	**10**	Ageloxime D	3.0262	−5.0562
	**11**	Maipomycin A		
	**12**	Isonaamine D	**2.4565**	**2.5205**
	**13**	Isonaamidine A	**1.6476**	**4.386**
	**14**	2,2-*bis*(6-bromo-1*H*-indol-3-yl)ethanamine	4.1514	−1.8628
	**15**	2,2-*bis*(6-fluoro-1*H*-indol-3-yl)ethanamine	2.9026	−1.4128
	**16**	Makaluvamine A	**−0.3414**	**3.1635**
	**17**	Makaluvamine F	**2.2402**	**2.5254**
	**18**	Makaluvamine G	**1.0552**	**3.189**
	**19**	Meridianin D	2.3034	−2.0575
	**20**	Collismycin C	1.3629	−1.2477
Terpenoids	**21**	α-bisabolol	4.4711	−1.4665
	**22**	Dolabellanes	5.4304	−3.5032
	**23**		4.0308	−1.2618
	**24**		5.0526	−1.8279
	**25**	Dictyol C	4.0017	−1.8996
	**26**	Dictyol L	1.1555	−2.9689
	**27**	Knightal	7.087	−20.275
	**28**	11(*R*)-hydroxy-12(20)-en-knightal	5.0026	−20.636
	**29**	11(*R*)-hydroxy-12(20)-en-knightol acetate	5.4872	−16.924
	**30**	Phorbaketal B	5.0073	−0.61496
	**31**	Phorbaketal C	5.0073	−0.61496
	**32**	Ophiobolin K	5.5062	0.094351
	**33**	6-*epi*-ophiobolin K	5.5062	0.094351
	**34**	6-*epi*-ophiobolin G	6.3296	−3.2017
	**35**	Siphonocholin	7.4008	−8.1908
	**36**	Halistanol sulfate A	1.5225	−5.4372
	**37**	5-episinuleptolide	1.6808	−17.833
	**38**	5-octylfuran-2(5*H*)-one	3.2099	−21.892
Fatty acids and derivatives	**39**	(9*Z*)-9-octadecenal	6.8564	−26.022
	**40**	Arachic acid	7.8801	−25.216
	**41**	Erucic acid	8.5367	−28.971
	**42**	(13*Z*)-13-octadecenale	6.8564	−17.802
	**43**	Tetracosanoic acid	9.6977	−25.216
	**44**	4-Phenylbutanoic acid	2.0516	−6.2653
	**45**	Stearidonic acid (18:4 n-3)	5.9625	−19.501
	**46**	Eicosapentaenoic acid (20:5 n-3)	6.6191	−14.291
	**47**	Docosapentaenoic acid (22:5 n-3)	7.5279	−20.741
	**48**	Docosahexaenoic acid (22:6 n-3)	7.2757	−10.83
	**49**	Mevalonolactone	−0.2323	−0.032673
	**50**	Myristic acid	5.1537	−25.216
	**51**	Oleic acid	6.7191	−28.971
	**52**	Lyngbyoic acid	3.9235	−18.267
	**53**	Benderadienne	6.2758	−26.52
	**54**	Pentadecanal	5.7454	−22.307
Polysaccharides	**55**	Fucoidan	−2.6337	−0.043172
	**56**	MO245	NA	NA
	**57**	Monomeric units of α-d-galactopyranosyl-(1→2)-glycerol-phosphate	NA	NA
Polyketides	**58**	Hygrocin C	**2.9757**	**2.234**
	**59**	Secalonic acid D	1.2992	−1.54
	**60**	Tetracenomycin D	3.1889	−1.1275
	**61**	Resistomycin	3.7044	−3.2806
	**62**	Resistoflavin	2.2781	−1.5295

Our review of marine molecules with anti-biofilm activity shows that many teams have discovered anti-biofilm extracts or molecules with bactericidal activity, while others have failed to mention them in detail. If a molecule has a bactericidal effect, it can de facto prevent the appearance of biofilm, but it may not have a curative effect. To truly speak of an anti-biofilm effect, it would be necessary to systematically define whether the dose used has an antibacterial effect, both in preventing and in curing pre-formed biofilm.

One of the major difficulties in research in this field is purification. In fact, many extracts lose their activity after purification. The quantities of molecules extracted may be too small to perform the necessary tests, the interaction between several molecules may be essential, or unsuitable solvents may be used. The importance of culture media is paramount in the production of molecules of interest. In the case of bacterial production, we have seen a very wide variety of media used, making it difficult to harmonize results and predict the type of molecules produced.

Methods for assessing biofilm formation are varied, with some teams using microplates to form a biofilm at the air–liquid interface, while others assess biofilms formed in microfluidics, magnetic beads, or plots. With such a wide variety of media, techniques, and solvents used for purification, there are countless opportunities for discovery or, conversely, lack of discovery.

The standardization required to harmonize results seems difficult to achieve, except perhaps in a large company, but it not be desirable, because it would ultimately limit the discoveries that fundamental science has to offer.

All these data show that there is still a lot of work to be done on marine anti-biofilm molecules and that this field has significantly evolved over the last 15 years.

## 4. Conclusions

Antibiotics are currently the main therapeutic solution used to combat bacterial infections. However, their massive and abusive use over the last 60 years has led to the development of multi-resistant bacteria, which are found all over the world, regardless of species.

The presence of bacteria in the form of biofilms leads to chronic and persistent infections, which in turn leads to the massive use of antibiotics. There is therefore an urgent need to find molecules with anti-biofilm activity that would limit their formation and help the immune system to fight the infection.

As shown above, natural marine products are a major source of metabolites with original skeletons, many of which have yet to be discovered. These secondary metabolites are an important source of potential drug candidates. By linking the various disciplines of fundamental research such as analytical chemistry, organic chemistry, and microbiology with knowledge of ecosystems, particularly chemical ecology, it becomes easier to find molecules of interest. The risk of rediscovery is always present, but has been reduced by the emergence of various techniques derived from analytical chemistry, such as metabolomics and the use of molecular networks.

This review highlighted the importance of distinguishing strict anti-biofilm molecules or extracts from those with antibacterial activities. It is astonishing to find only around sixty strict anti-biofilm molecules over more than a decade. Combining anti-biofilm and bactericidal tests is therefore of real importance. Bacteria and fungi appear to be interesting sources in the field of anti-biofilm molecules, not only because of the durability of their source, but also because of the possibility of accessing their genomes. Indeed, biosynthetic pathways of molecules of interest can therefore be studied, allowing an improvement in their production through biotechnological engineering.

The druggable aspect is interesting, but should not put an end to studies on less druggable molecules, given the subsequent possibilities for galenic formulation to improve bioavailability.

It is already possible to observe promising molecules showing activity on highly problematic multi-resistant bacteria such as *S. aureus* and *P*. *aeruginosa*, without showing any activity on the growth of planktonic bacteria. The information that is generally lacking relates more to the modes of action of these molecules, which can be very wide-ranging.

These compounds, which are generally active at low concentrations, should have negligible or no side-effects on patients, animals, or the environment, and should make it possible to limit antibiotic resistance linked to selection pressure.

It would be interesting to work on the terminology of the term “anti-biofilm” and add categories according to the mode of action of the molecules, if this is known. Furthermore, the distinction between antibiotics and anti-biofilms seems essential at a time when antibiotic resistance is such a major issue. A biocidal activity test would therefore seem to be an essential prerequisite for any research into anti-biofilm activity.

## Figures and Tables

**Table 1:** Non-biocidal anti-biofilm molecules reported in the literature. Compounds are grouped by family, and for each one the producing organism and target organism(s) are indicated. When modes of action are known or assumed, they are described at the end of the table. Each type of organism is distinguished by the color associated with the box (Producer organisms: **bacteria**, **fungi**, **sponge**, **cnidarian**, **alga**, **other invertebrate**).

Compound Family	Compound Number	Compound Name	Producing Organisms	Target Organisms	Mechanisms of Action	Reference
Peptides and proteins	Unknown structure	Cyclic lipopeptide	*Pseudomonas* sp. TAD1S	*S. aureus*	Surfactant	[35]
	Unknown structure	Alterocin	*Pseudoalteromonas* sp. 3J6	*P. aeruginosa; E. coli; S. enterica; Vibrio* sp. D01; *Paracoccus* sp. 4M6	Impact on bacterial adhesion	[36,37,38]
	Unknown structure	P004	*Pseudoalteromonas* sp. IIIA004	*Roseovarius* sp. VA014		[39]
	**1**	*cis*-cyclo(Leucyl-Tyrosyl)	Sponge associated *Penicllium* sp.	*S. epidermidis*		[40]
	Unknown structure	Scyreprocin	*Scylla paramamosain*	*Candida albicans* and *C. neoformans*		[41]
	**2**	Paracentrin 1	*Paracentrotus lividus*	*S. epidermidis* DSM 3269; *S. aureus* ATCC 29213; *P. aeruginosa*		[42,43,44]
	Unknown structure	Catasan	*Psychrobacter* sp. TAE2020	*S. epidermidis* RP62A	Reduces biofilm biomass and modifies its structure	[45]
	**3**	Nesfactin	*Nesterenkonia* sp. MAS31 isolated from *Fasciospongia cavernosa*	*P. aeruginosa*	Quenches QS via LasR	[46]
	**4**	Cyclo(*L*-Trp-*L*-Ser)	*Rheinheimera aquimaris*	*Chromobacterium violaceum* and *P. aeruginosa* PAO1	Decreases production of violacein, exhibits pyocyanin production, swimming motility, adhesion, and biofilm formation	[47]
Phenolic compounds	**5**	2,4-di-tert-butylphenol	*Vibrio alginolyticus* G16	*S. marcescens*	Impacts production of virulence factor via QS	[48]
	**6**	Methyl benzoate	*Pseudomonas aeruginosa* CBMGL12 isolated from coral *Favites* sp.	*S. aureus* MTCC96	Diminishes virulence and biofilm phenotypes, seems to target the QS	[49]
	**7**	Methyl phenylacetate				
Alkaloids	**8**	Psammaplin A	*Aplysinella rhax*	*P. aeruginosa*	Inhibits production of elastase and QS	[50]
	**9**	Bisaprasin				
	**10**	Ageloxime D	*Agelas nakamurai*	*S. epidermidis*		[51]
	**11**	Maipomycin A	*Kibdelosporangium phytohabitans* XY-R10	*Actinobacter baumannii* and *P. aeruginosa*	Iron chelator	[52]
	**12**	Isonaamine D	*Leucetta chagosensis*	*V. harveyi*	Inhibitor activity on all three QS pathways	[53]
	**13**	Isonaamidine A				
	**14**	2,2-*bis*(6-bromo-1*H*-indol-3-yl)ethanamine	*Didemnum candidum*, and *Orina* spp.	*S. aureus* CH 10850 and *S. aureus* ATCC 29213		[54,55]
	**15**	2,2-*bis*(6-fluoro-1*H*-indol-3-yl)ethanamine				
	**16**	Makaluvamine A	*Zyzzya fuliginosa*	*Streptococcus mutans*		[56]
	**17**	Makaluvamine F				
	**18**	Mavaluvamine G	*Histodermella* sp.			
	**19**	Meridianin D	*Aplidium meridianum*	*S. aureus*		[57,58]
	**20**	Collismycin C	*Streptomyces* sp. MC025	*S. aureus*		[59]
Terpenoids	**21**	α-bisabolol	*Padina gymnospora*	*Serratia marcescens*	Inhibits prodigiosin and protease production, and acts on bacterial motility and hemolysin production	[60]
	**22**	Dolabellanes	*Dictyota* sp.	*Pseudoalteromonas* sp.		[61]
	**23**					
	**24**					
	**25**	Dictyol C				
	**26**	Dictyol L	*Dictyota pinnatifida*	*P. aeruginosa*		[62]
	**27**	Knightal	*Eunicea knighti*	*Chromobacterium violaceum*, *S. aureus*, *V. harveyi* and *P. aeruginosa*	Anti-QS activity	[63,64]
	**28**	11(*R*)-hydroxy-12(20)-en-knightal				
	**29**	11(*R*)-hydroxy-12(20)-en-knightol acetate				
	**30**	Phorbaketal B	*Phorbas* sp.	*S. aureus*	Inhibition in expression of the biofilm-related hemolysin gene *hla* and the *nuc1* nuclease gene	[65]
	**31**	Phorbaketal C				
	**32**	Ophiobolin K	*Emericella variecolor*	*Mycobacterium smegmatis*		[66]
	**33**	6-*epi*-ophiobolin K				
	**34**	6-*epi*-ophiobolin G				
	**35**	Siphonocholin	*Siphonochalina siphonella*	*C. violaceum* and *P. aeruginosa*	Altered production of elastase, total protease, pyocyanin, chitinase and exopolysaccharides	[67]
	**36**	Halistanol sulfate A	*Petromica ciocalyptoides*	*S. mutans*		[68]
	**37**	5-episinuleptolide	*Sinularia leptoclados*	*A. baumannii* ATCC 19606, BAA747, 29115, 68704, D4	Diminish production of the extracellular polysaccharide poly-β-(1,6)-N-acetylglucosamine (PNAG)	[69]
	**38**	5-octylfuran-2(5H)-one	*Streptomyces* sp.	*E. coli* K12, *P. aeruginosa* PAO1 and methicillin-resistant *Staphylococcus aureus*	Matrix destruction and interference with AI-2 mediated QS system	[70]
Fatty acids and derivatives	**39**	(9Z)-9-octadecenal	*Streptomyces griseoincarnatus* HK 12	*S. aureus* and *P. aeruginosa*	(13Z)-13-octadecenal is thought to target the quorum sensing system by binding 3-oxo-C12 HSL in *P. aeruginosa*	[71]
	**40**	Arachic acid				
	**41**	Erucic acid				
	**42**	(13Z)-13-octadecenale				
	**43**	Tetracosanoic acid				
	**44**	4-Phenylbutanoic acid	*Bacillus pumilus* S6-15	*P. aeruginosa*, *B. indicus* MTCC5559 and *B. pumilus* MTCC5560		[72,73]
	**45**	Stearidonic acid (18:4 n-3)	Various marine origins	*Candida albicans* and *C. dubliniensis*	Oxidative stress	[74]
	**46**	Eicosapentaenoic acid (20:5 n-3)				
	**47**	Docosapentaenoic acid (22:5 n-3)				
	**48**	Docosahexaenoic acid (22:6 n-3)				
	**49**	Mevalonolactone	*Sordariales associated to Mycale magnirhaphidifera*	*S. epidermidis*		[75]
	**50**	Myristic acid	*Mycale contarenii*	*S. aureus* methicillin susceptible and resistant, *L. monocytogenes*	Repress transcription of *fnbA* and *fnbB* genes, fibronectin-binding protein, and *icaADBC* operon (polysaccharide intercellular adhesin)	[76]
	**51**	Oleic acid				
	**52**	Lyngbyoic acid	*Lyngbya* sp.	*P. aeruginosa* PaO1	Inhibits biofilm formation (biovolume) and QS pathways	[77]
	**53**	Benderadienne				
	**54**	Pentadecanal	*P. haloplanktis* TAC125	*S. epidermidis*	Impair biofilm formation	[31]
Polysaccharides	Unknown structure	A101	*Vibrio* sp. QY101	Wide range of Gram positive and negative		[78]
	**55**	Fucoidan	*Fucus vesiculosus*	*S. mutans* and *S. sobrinus*	Only active on biofilm formation	[79]
	**56**	MO245	*Vibrio alginolyticus* sp.	*P. aeruginosa* PaO1 and *V. harveyi* DSM19623	Leads to abiotic and bacterial surface modification	[80]
	**57**	Monomeric units of α-D-galactopyranosyl-(1→2)-glycerol-phosphate (1800 kDa)	*B. licheniformis* associated with *Spongia officinalis*	*E. coli* PHL628, *P. fluorescences*	Reduces cell surface hydrophobicity	[81]
Polyketides	**58**	Hygrocin C	*Streptomyces* sp. SCSGAA 0027	*S. aureus* and *B. amyloliquefaciens* SCSGAB0082	Reduces matrix formation, decreases surface hydrophobicity, impacts on bacterial flagellar system	[82]
	**59**	Secalonic acid D	*Penicillium* sp. SCSGAF0023 (CCTCC M 2012507)	*S. aureus*	Targets genes associated to biofilm formation: *agr*, *isaA*, *icaA*, and *icaD*	[83]
	**60**	Tetracenomycin D	*Streptomyces* sp. EG1	*S. aureus* and *E. coli*	Target biofilm forming protein (ClfB in *S. aureus* and CSgG in *E. coli*)	[84]
	**61**	Resistomycin				
	**62**	Resistoflavin				
